# Risk factors for severe immune‐related pneumonitis after nivolumab plus ipilimumab therapy for non‐small cell lung cancer

**DOI:** 10.1111/1759-7714.15385

**Published:** 2024-06-03

**Authors:** Toshiyuki Sumi, Motoki Sekikawa, Yuta Koshino, Daiki Nagayama, Yuta Nagahisa, Keigo Matsuura, Naoki Shijubou, Koki Kamada, Keito Suzuki, Takumi Ikeda, Haruhiko Michimata, Hiroki Watanabe, Yuichi Yamada, Koichi Osuda, Yusuke Tanaka, Hirofumi Chiba

**Affiliations:** ^1^ Department of Pulmonary Medicine Hakodate Goryoukaku Hospital Hakodate Japan; ^2^ Department of Respiratory Medicine and Allergology Sapporo Medical University School of Medicine Sapporo Japan; ^3^ Division of Radiology Hakodate Goryoukaku Hospital Hakodate Japan

**Keywords:** immune checkpoint inhibitor, non‐small cell lung cancer, pneumonitis, surfactant protein D

## Abstract

**Background:**

The efficacy of anti‐CTLA‐4 antibody (ipilimumab) plus anti‐programmed cell death 1 antibody (nivolumab) in treating advanced non‐small cell lung cancer (NSCLC) is impeded by an elevated risk of severe immune‐related adverse events. However, our understanding of associations among pre‐existing fibrosis, emphysematous changes, and objective indicators as predictive factors is limited for severe pneumonitis in NSCLC patients receiving this combination therapy. Thus, we retrospectively investigated these associations, including overall tumor burden, before treatment initiation in the Japanese population.

**Methods:**

We focused on patients (*n* = 76) with pre‐existing interstitial lung disease (ILD) to identify predictors of severe pneumonitis. Variables included age, sex, smoking status, programmed cell death ligand 1 expression, overall tumor burden, chest computed tomography‐confirmed fibrosis, serum markers, and respiratory function test results.

**Results:**

Severe pneumonitis was more frequent in patients with squamous cell carcinoma, fibrosis, low diffusing capacity for carbon monoxide (%DLCO), and high surfactant protein D (SP‐D) level. Notably, squamous cell carcinoma, baseline %DLCO, and SP‐D level were significant risk factors. Our findings revealed the nonsignificance of tumor burden (≥85 mm) in predicting severe pneumonitis, emphasizing the importance of pre‐existing ILD. Conversely, in cases without pre‐existing fibrosis, severe pneumonitis was not associated with %DLCO or SP‐D level (93.2% vs. 91.9%, and 63.3 vs. 40.9 ng/mL, respectively) and was more common in patients with a large overall tumor burden (97.5 vs. 70.0 mm).

**Conclusion:**

Vigilant monitoring and early intervention are crucial for patients with squamous cell carcinoma, high SP‐D level, or low %DLCO undergoing ipilimumab plus nivolumab therapy.

## Graphical abstract

We investigated the relationship between tumor burden and the risk of severe pneumonitis in patients with non‐small cell lung cancer (NSCLC) treated with ipilimumab and nivolumab. The study, conducted from December 2020 to December 2022 with 76 NSCLC patients, evaluated various factors including age, sex, smoking status, PD‐L1 expression, tumor size, presence of fibrosis, serum markers, and respiratory function tests. The results indicate that lower diffusing capacity for carbon monoxide (%DLCO), higher surfactant protein D (SP‐D) levels, and the presence of squamous cell carcinoma along with fibrosis significantly increase the risk of severe pneumonitis, with respective odds ratios indicating substantial increases in risk. Additionally, the study found that in patients without pre‐existing fibrosis, an increase in tumor burden correlated significantly with the incidence of severe pneumonitis. These findings emphasize the need for careful monitoring and proactive interventions in at‐risk patients, illustrating the critical influence of tumor characteristics and treatment modalities on patient outcomes in NSCLC therapy.

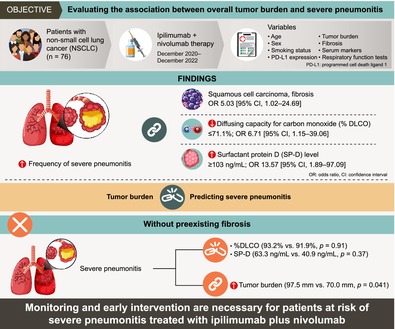

## INTRODUCTION

Recently, the combination of an anti‐CTLA‐4 antibody (ipilimumab) and an anti‐programmed cell death 1 (PD‐1) antibody (nivolumab) has been reported to improve the prognosis of patients with previously untreated advanced non‐small cell lung cancer (NSCLC) in comparison to platinum combination treatment, as evidenced in the CheckMate227 and CheckMate9LA trials.^1^, ^2^ However, ipilimumab plus nivolumab therapy is linked to a higher rate of immune‐related adverse events (irAEs) that anti‐PD‐1 or anti‐programmed cell death ligand 1 (PD‐L1) antibody monotherapy. Pneumonitis, recognized as a particularly severe irAE, poses a substantial risk of becoming life‐threatening in severe cases. The reported incidence of pneumonitis in patients with advanced NSCLC treated with anti‐PD‐1 monotherapy is 3%–5%,^3^, ^4^, ^5^, ^6^ whereas the frequency escalates to 5%–7%^1^, ^2^ in those receiving to ipilimumab plus nivolumab, reaching over 10% in the Japanese population.^7^

The association between the presence of pre‐existing fibrosis and/or emphysematous changes and the development of pneumonitis remains unclear owing to the general exclusion of patients with interstitial lung disease (ILD) from prospective clinical trials. A previous intervention trial involving immune checkpoint inhibitors (ICI) in patients with ILD revealed a notably higher incidence of pneumonitis in patients with honeycomb lungs.^8^ Conversely, it was less common in those with mild ILD, excluding risk factors such as fibrosis, autoimmune disease, and decreased vital capacity percentage.^9^ Retrospective studies have indicated that pre‐existing fibrosis, assessed using computed tomography (CT) before commencing ICI treatment, is associated with pneumonitis development.^10^ However, the challenge lies in the difficulty of generalizing the evaluation and quantification of fibrosis to clinical practice, given the substantial variations in approaches among different physicians.^11^, ^12^

Objective indicators of interstitial lung injury include serum markers and respiratory function test results, with KL‐6 and surfactant protein D (SP‐D) serum markers identified as specific markers of ILD.^13^, ^14^ Elevated levels of these markers are often observed in drug‐induced lung injuries, offering diagnostic assistance and aiding in disease monitoring.^15^, ^16^, ^17^, ^18^ Additionally, SP‐D has been utilized to distinguish between bacterial and drug‐induced pneumonia.^16^ Limited research exists on whether KL‐6 or SP‐D can predict drug‐induced lung injury before initiating treatment. Furthermore, respiratory function tests play a crucial role in assessing drug‐induced lung injury, with decreased diffusing capacity for carbon monoxide (DLCO) serving as a precursor to such injuries and forced vital capacity (FVC) correlating with the progression of interstitial lung injury.^19^, ^20^ However, evidence supporting pulmonary function tests as predictors of drug‐induced lung injury remains insufficient.

Pneumonitis is associated with a higher treatment discontinuation rate than other irAEs,^21^ and the severity of pneumonitis significantly influences treatment continuation and prognosis. Therefore, predicting the development of severe pneumonitis before treatment initiation and intervening promptly to prevent its escalation are highly desirable. However, studies on the prediction of pneumonitis onset and severity in patients receiving ipilimumab plus nivolumab treatment are limited. We previously reported an association between grade 3 or higher severe irAEs, including pneumonitis, and overall tumor burden before treatment initiation with ipilimumab plus nivolumab therapy.^22^ However, we did not examine the correlation between high or low overall tumor burden and the development of severe pneumonitis.

In this study, we aimed to explore the association between overall tumor burden and severe pneumonitis development. To this end, we retrospectively investigated the association of severe pneumonitis development in patients with NSCLC treated with ipilimumab plus nivolumab, utilizing clinical data (such as pre‐existing fibrosis/emphysema, serum markers, and respiratory function tests) obtained before treatment.

## METHODS

### Patient selection and data collection

Data of consecutive patients with advanced NSCLC who received treatment with nivolumab plus ipilimumab, with or without chemotherapy (NIVO + IPI ± chemo), between December 2020 and December 2022 were retrospectively evaluated. Data were extracted from the prescription drug database of Hakodate Goryoukaku Hospital, and only patients with measurable disease, respiratory function tests, and pretreatment measurements of SP‐D and KL‐6 were included. Follow‐up was continued until the end of January 2023.

The opt‐out method was used to obtain informed consent from the patients, and comprehensive information about the research was made available on the hospital facility and website. Institutional Ethics Committee approval (approval no. 2023‐004) was obtained on January 20, 2023, and the study adhered to the principles outlined in the Declaration of Helsinki.

### Patient characteristics

Data on variables such as age, sex, Eastern Cooperative Oncology Group performance status, smoking status, histopathology, disease stage, PD‐L1 expression, chemotherapy add‐on, chest imaging findings (CT conducted within 4 weeks of treatment initiation), pretreatment respiratory function tests, pretreatment SP‐D and KL‐6 levels, treatment response (best response), progression‐free survival (PFS), pneumonitis (onset date and worst grade value), and tumor burden were extracted from the database.

### Definition and assessment of severe pneumonitis

Treatment response was determined according to the Response Evaluation Criteria in Solid Tumors, version 1.1 (RECIST version 1.1). Severe pneumonitis was defined as toxicities related to ICI therapy (grade ≥3 according to the Common Terminology Criteria for Adverse Events, version 5.0) or adverse effects following which the continuation of treatment was considered impossible by physicians. Baseline overall tumor burden was measured using CT or magnetic resonance imaging according to RECIST version 1.1, based on a previous study.^23^ Tumor burden was defined as the sum of the longest diameters of a maximum of five target lesions and up to two lesions per organ. PD‐L1 expression was determined using the Dako PD‐L1 immunohistochemistry (IHC) 22C3 PharmDx test according to the manufacturer's instructions (SRL Inc., Tokyo, Japan).^24^

### Respiratory function and imaging analysis

Baseline chest imaging was performed using CT to quantify the emphysematous and interstitial changes in the lungs. Quantification was performed by two respiratory specialists following the protocols described previously.^10^ The fibrosis score was defined as follows: score 0, no fibrosis; score 1, interlobular septal thickening: no honeycomb; score 2, honeycomb (with/without septal thickening) < 25% of the lobe; and score 3, honeycomb (with/without septal thickening) 25%–49% of the lobe. The emphysema score was defined as follows: score 0, no low‐attenuation areas (LAAs); score 1, sparse, scattered small LAAs up to 5 mm in diameter; score 2, adjacent LAAs up to 10 mm in diameter; and score 3, LAAs >10 mm adjacent to or indistinguishable from each other (Figure S1).

### Statistical analysis

Time to events was estimated using the Kaplan–Meier method, and a log‐rank test was employed to compare survival curves among different groups. Comparisons between the groups were performed using the Mann–Whitney U and Fisher's exact tests for continuous and categorical variables, respectively. Receiver operating characteristic (ROC) curve analysis, with tumor burden as an independent variable and the occurrence or nonoccurrence of severe irAEs as bivariate variables, was performed to determine the cutoff level of tumor burden. Youden's index was used to determine the optimal cutoff value. Odds ratio (OR) was evaluated using Fisher's exact test and using multivariate logistic regression in multivariate analysis. Data are presented as median and interquartile range (IQR). Statistical significance was set at *p* < 0.05, and all analyses were executed using EZR, a graphical user interface for R software (The R Foundation for Statistical Computing, Vienna, Austria)^25^ and GraphPad Prism 10 (GraphPad Software, USA).

## RESULTS

### Patient inclusion and characteristics

**FIGURE 1:**
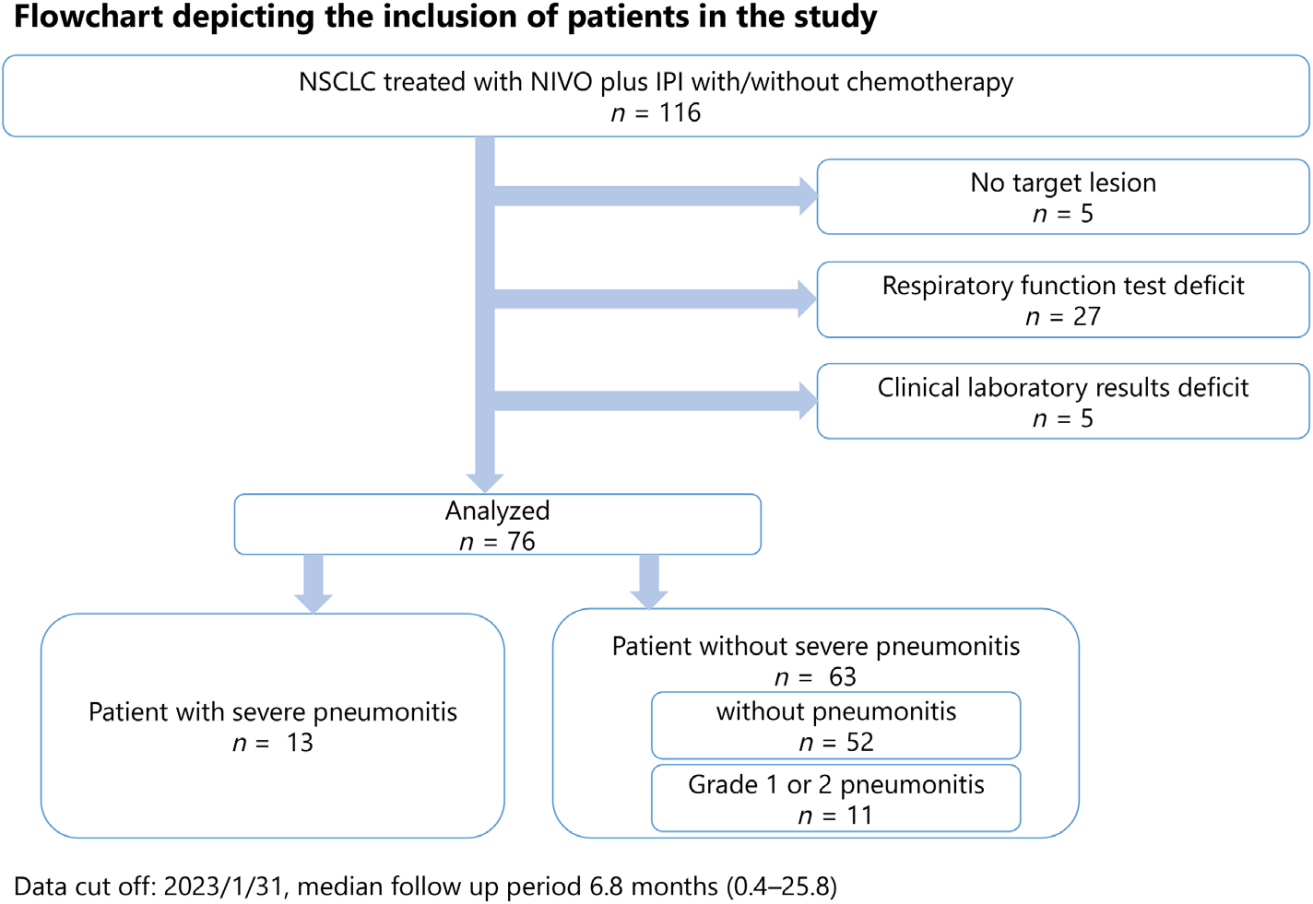
Flow chart depicting patient enrollment. IPI, ipilimumab; NIVO, nivolumab; NSCLC, non‐small cell lung carcinoma.

A total of 116 patients who received nivolumab plus ipilimumab with or without chemotherapy for advanced NSCLC were identified. However, five patients were excluded because their lesions could not be measured, 27 patients were excluded owing to a lack of respiratory function test data, and five patients were excluded owing to a lack of SP‐D and KL‐6 data. Overall, 76 patients were included in the study (Figure 1), and their characteristics are summarized in Table 1. Of the patients included in this study, 70/76 had a history of smoking, and 15/21 patients (71.4%) with an F score of ≥1 at baseline had emphysematous complications with an E score of ≥1.

**TABLE 1:** Patient characteristics.

Factor	Total (*n* = 76)	Patients with severe pneumonitis (*n* = 13)	Patients without severe pneumonitis (*n* = 63)	*p*‐value^a^
Age (years)				
≥75		5	28	0.766
<75		8	35	
Sex				
Female		3	18	1
Male		10	45	
ECOG PS				
0		6	42	0.34
1		7	20	
2		0	1	
Stage				
III		5	15	0.609
IV		7	39	
Rec		1	9	
PD‐L1				
≥50%		4	22	0.287
1%–49%		8	25	
<1%		1	16	
Regimen				
NIVO + IPI		6	29	1
NIVO + IPI + chemo		7	34	
Best of response				
CR		0	1	0.93
PR		8	34	
SD		3	18	
Progressive disease		2	10	
Histology				
NSQ		3	36	0.034
SQ		10	27	
E score				
≥1		9	29	0.222
F score				
≥1		7	14	0.037
%FEV1	Median [IQR]	71.21 [56.11–77.44]	74.14 [64.35–77.73]	0.581
%FVC	Median [IQR]	87.70 [72.50–89.50]	88.80 [75.00–99.00]	0.424
%DLCO	Median [IQR]	58.80 [50.80–91.90]	91.30 [78.00–109.80]	<0.01
KL‐6	Median [IQR]	468.00 [341.00–536.00]	374.00 [279.00–551.00]	0.44
SP‐D	Median [IQR]	125.00 [66.00–156.00]	51.60 [30.25–87.50]	<0.01
Overall tumor burden (RECIST)	Median [IQR]	80.00 [65.00–100.00]	70.00 [42.00–90.00]	0.206

Abbreviations: %DLCO, percent predicted diffusing capacity for carbon monoxide; %FEV, percent predicted forced vital capacity; %FEV1, percent predicted forced expiratory volume in one second; chemo, chemotherapy; CR, complete response; E score, emphysema score; ECOG PS, Eastern Cooperative Oncology Group performance status; F score, fibrosis score; IPI, ipilimumab; IQR, interquartile range; NIVO, nivolumab; NSQ, nonsquamous cell carcinoma; PD‐L1, programmed cell death ligand 1; PR, partial response; RECIST, Response Evaluation Criteria in Solid Tumors; SD, stable disease; SP‐D, surfactant protein D; SQ, squamous cell carcinoma.

^a^ Fisher's exact tests or Mann–Whitney U test.

### Pneumonitis incidence and severity

In the entire study population, 24 developed pneumonitis of variable grades, of whom 13 developed severe pneumonitis. Severe pneumonitis was more frequent in patients with squamous cell carcinoma (SQ), F score ≥1, low %DLCO, and high SP‐D level. A summary of patients with pneumonitis among all patients is presented in Table S1. The number of patients with pneumonitis of grades 1, 2, 3, 4, and 5 was 0, 11, 11, 1, and 1, respectively. The patient with grade 5 tested positive for the COVID‐19 antigen at the time of onset. The median time from the start of treatment to the onset of pneumonitis was significantly shorter for patients with severe pneumonitis than for those with mild pneumonitis (grade 1 or 2; 27 days, interquartile range [IQR] = 15–61 vs. 112 days, IQR = 41–150.5, *p* = 0.03; Figure S2).

### Fibrosis, emphysema, and pneumonitis scores

The scores for fibrosis, emphysema, and pneumonitis grade are summarized in Table 2. The F score was ≥1 for 21/76 patients, and 38/76 patients had an E score of ≥1. The incidence of grade 3 or higher pneumonitis with F score ≥1 and E score ≥1 was 16.7% and 8.7%, respectively, whereas patients with combined fibrosis (≥1) and emphysema (≥1) had a high incidence of grade 3 or higher pneumonitis (40%).

**TABLE 2:** Incidence of nivolumab plus ipilimumab‐related pneumonitis.

	Total, *n*	Any grade, *n* (%)	Grade ≥3, *n* (%)
Total	76	24 (31.6)	13 (17.1)
Fibrosis score on baseline CT			
0	55	12 (21.8)	6 (10.9)
≥1	21	12 (57.1)	7 (33.3)
1	14	9 (64.3)	4 (28.6)
2	5	1 (20.0)	1 (20.0)
3	2	2 (100.0)	2 (100.0)
Emphysema score on baseline CT			
0	38	7 (18.4)	4 (10.5)
≥1	38	19 (50.0)	9 (23.7)
1	21	7 (33.3)	3 (14.3)
2	14	9 (64.3)	5 (35.7)
3	3	1 (33.3)	1 (33.3)
Category of CT findings			
No fibrosis and emphysema	32	4 (12.5)	1 (3.1)
Fibrosis alone (≥1)	6	3 (50.0)	1 (16.7)
Emphysema alone (≥1)	23	8 (34.8)	2 (8.7)
Combined fibrosis (≥1) and emphysema (≥1)	15	9 (60.0)	6 (40.0)
Combined fibrosis (≥2) and emphysema (≥2)	3	3 (100.0)	3 (100.0)

Abbreviation: CT, computed tomography.

### Biomarker and DLCO analyses

**FIGURE 2:**
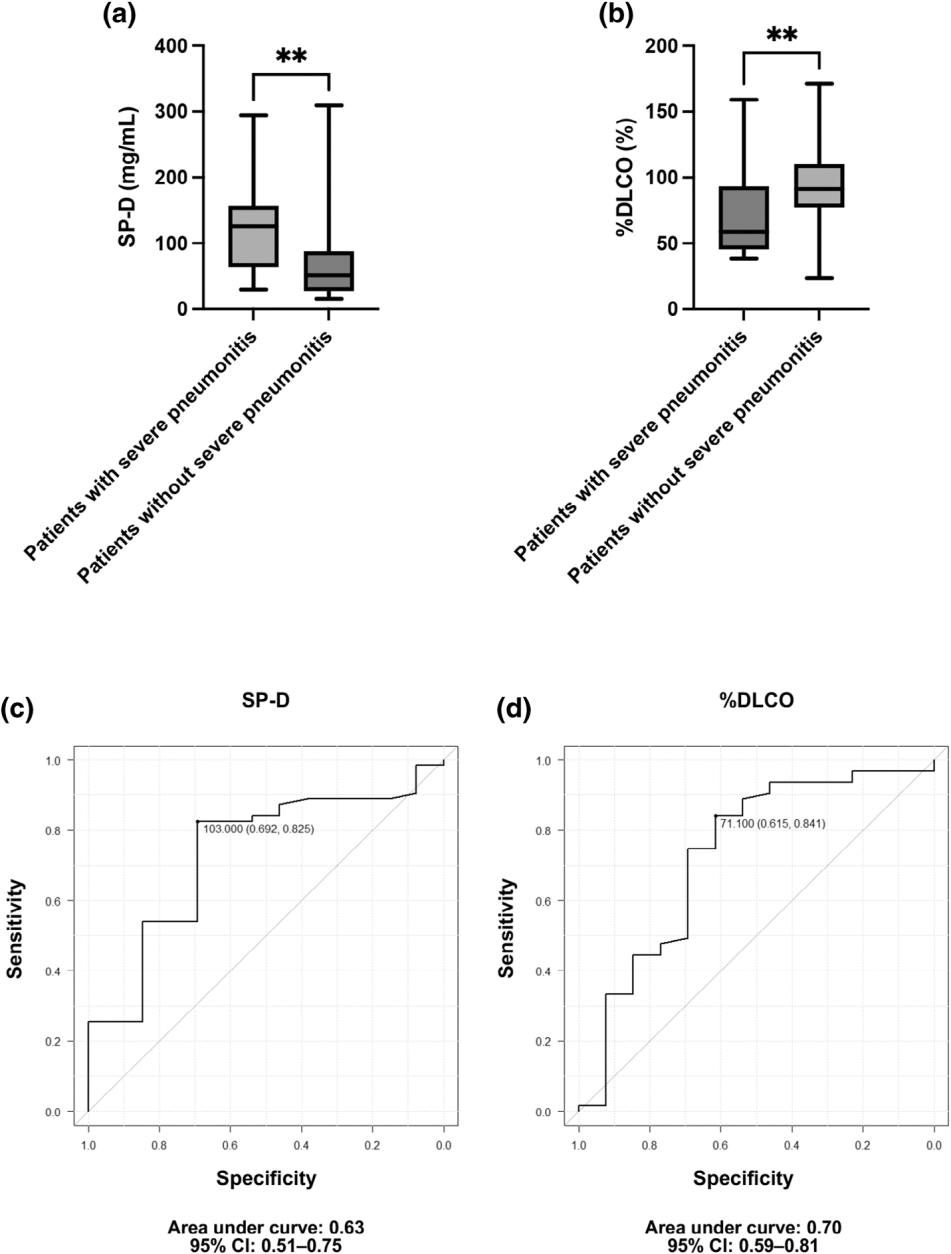
Box plot showing the relationship of severe pneumonitis with (a) surfactant protein D (SP‐D) level and (b) percent predicted diffusing capacity for carbon monoxide (%DLCO). (c) Receiver operating characteristic curve analysis of severe pneumonitis to determine the cutoff values for SP‐D and (d) %DLCO. CI, confidence interval; TPS, tumor proportion score. **p < 0.01.

SP‐D and %DLCO levels differed significantly between patients with and those without severe pneumonitis (125 ng/mL, IQR = 66.0–156.0 vs. 51.6 ng/mL, IQR = 30.3–87.5, *p* = 0.0079; Figure 2a, and 58.8%, IQR = 50.8%–91.9% vs. 91.3%, IQR = 78.0–109.8, *p* = 0.0087; Figure 2b, respectively). The ROC curve analyses indicated that the optimal cutoff values for SP‐D and %DLCO were 103.0 (sensitivity = 74.5%, specificity = 53.3%, area under the curve = 0.63, 95% confidence interval [CI]: 0.51–0.75; Figure 2c) and 71.1% (sensitivity = 73.3%, specificity = 66.0%, area under the curve = 0.70, 95% CI: 0.59–0.81; Figure 2d), respectively. Among 15 patients with combined fibrosis (≥1) and emphysema (≥1), the SP‐D level was above the cutoff in nine (60.0%) and %DLCO was above the cutoff in eight (53.3%); all eight patients whose levels exceeded the cutoff value for %DLCO exceeded the cutoff value for SP‐D.

### Univariate and multivariate analyses

Univariate and multivariate analyses were performed using pretreatment clinical parameters. Tumor burden was included in the multivariate analysis because it is associated with the risk of irAEs.^21^ SQ, low %DLCO, and high SP‐D level, but not F score ≥1 and tumor burden ≥85 mm, correlated with the onset of severe pneumonitis (Table 3). In the multivariate analysis, including all variables from all univariate analyses, the ORs of high SP‐D level, low %DLCO, and SQ for severe pneumonitis were 13.57 (95% CI: 1.89–97.09, *p* = 0.0094), 6.71 (95% CI: 1.15–39.06, *p* = 0.034), and 5.03 (95% CI: 1.02–24.69, *p* = 0.047), respectively.

**TABLE 3:** Risk factors for severe pneumonitis.

Variable	Patients with severe pneumonitis (*n* = 13)	Patient without severe pneumonitis (*n* = 63)	Univariate			Multivariate		
			Odds ratio	95% CI	*p*‐value	Odds ratio	95% CI	*p*‐value
Overall tumor burden ≥85 mm	6	21	1.71	0.47–5.57	0.53	1.05	0.20–5.49	0.96
F score ≥1	7	14	4.08	1.18–13.10	0.04	1.37	0.20–9.27	0.75
SQ	10	27	4.36	1.00–27.02	0.034	5.03	1.02–24.69	0.047
%DLCO ≤71.1	8	12	6.8	1.97–22.83	0.0036	6.71	1.15–39.06	0.034
SP‐D ≥103	9	12	9.65	2.70–30.62	0.0007	13.57	1.89–97.09	0.0094

Abbreviations: %DLCO, percentage predicted diffusing capacity for carbon monoxide; CI, confidence interval; F score, fibrosis score; SP‐D, surfactant protein D; SQ, squamous cell carcinoma.

### PFS and overall survival (OS)

**FIGURE 3:**
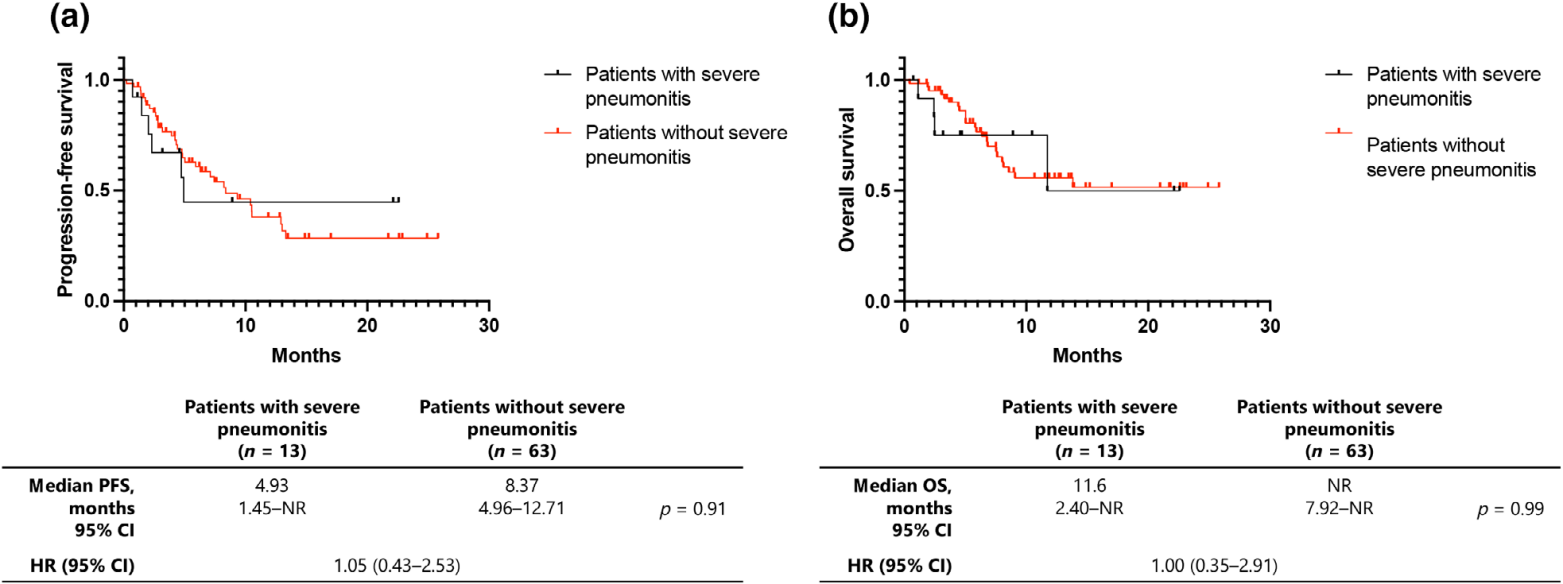
Kaplan–Meier curves of (a) progression‐free survival (PFS) and (b) overall survival (OS) in patients with non‐small cell lung cancer. CI, confidence interval; HR, hazard ratio.

The median PFS and OS did not significantly differ between patients with and those without severe pneumonitis (4.93 vs. 8.37 months, *p* = 0.91, hazard ratio [HR] = 1.05 [95% CI: 0.43–2.53] and 11.6 months vs. not reached, *p* = 0.99, HR = 1.00 [95% CI: 0.35–2.91], respectively) (Figure 3a,b). However, PFS was 4.93, 13.0, and 7.1 months for severe, mild, and without pneumonitis, respectively. The median OS was 11.6 months, not reached, and 13.6 months for severe, mild, and without pneumonitis, respectively. Patients with mild pneumonitis tended to have a longer PFS and OS (Figure S3a,b).

### Subpopulation analysis

We examined the relationship between severe pneumonitis and SP‐D level, %DLCO, and overall tumor burden in population with an F score of 0 (*n* = 55). Notably, in contrast to the overall population, we observed a significant difference in the SP‐D level and %DLCO between patients with severe pneumonitis and those without severe pneumonitis (63.3 ng/mL, IQR 39.7–110.3 vs. 40.9 ng/mL, IQR = 24.9–71.5, *p* = 0.37; Figure S4a, and 93.2%, IQR = 81.9–100.4 vs. 91.9%, IQR = 79.0–115.5, *p* = 0.91; Figure S4b, respectively), and the overall tumor burden was significantly higher in the severe pneumonitis group (97.5 mm, IQR = 83.8–115.0 vs. 70.0 mm, IQR = 45.0–90.0, *p* = 0.041; Figure S4c).

We further examined whether the risk factors for severe pneumonitis differed with or without the addition of platinum combination therapy to NIVO + IPI therapy. We performed univariate analysis of the factors used in the multivariate analysis of this study in the NIVO + IPI and NIVO + IPI + chemo groups (Table S2). The SP‐D level was the only factor that was significantly different between the groups; the F score and %DLCO were significantly associated with only NIVO + IPI + chemo.

Finally, we examined whether risk factors differed by the severity of pneumonia. Grouping all patients into those with any grade pneumonitis (*n* = 24) and those without pneumonitis (*n* = 52), we found that any grade pneumonitis was more frequent in patients with E score ≥1, F score >1, low %DLCO, and high SP‐D level (Table S3). On the contrary, classifying patients with any grade pneumonitis into those with severe pneumonitis (grade ≥3, *n* = 13) and those with mild pneumonitis (grade 1 or 2, *n* = 11), severe pneumonitis was more frequent only in patients with a high SP‐D level (Table S4).

## DISCUSSION

In this study, we identified SQ, baseline %DLCO, and SP‐D levels as potential risk factors for the development of severe pneumonitis in patients receiving first‐line nivolumab plus ipilimumab with or without chemotherapy. The risk factors for pneumonitis differ between pembrolizumab, which is widely used in the treatment of lung cancer, and nivolumab plus ipilimumab with or without chemotherapy. Previous studies have identified several risk factors for pneumonitis in patients with lung cancer treated with pembrolizumab monotherapy, including pre‐existing lung conditions, prior radiation therapy, older age, lower performance status, and higher PD‐L1 expression status.^26^, ^27^, ^28^, ^29^ These factors can influence the incidence and severity of pneumonitis. In this study, existing lung conditions, F score, %DLCO, and SP‐D level were identified as risk factors in patients receiving nivolumab plus ipilimumab with or without chemotherapy.

Factors associated with disease progression and mortality in ILD, such as pulmonary fibrosis, FVC, DLCO, KL‐6, and SP‐D, exhibit similarities.^30^ Even in the absence of interventions such as drugs or radiation, these factors act as poor prognostic indicators for ILD. Notably, FVC tends to be preserved in patients with combined pulmonary fibrosis and emphysema (CPFE),^31^, ^32^ potentially masking the decline in FVC attributable to fibrotic lesions. CPFE might explain the lack of FVC differences between patients with and those without severe pneumonitis. Conversely, DLCO serves as a diminishing parameter for both emphysema and fibrosis, possibly better reflecting the extent of pre‐existing interstitial lung injury, although potential overestimation occurs due to emphysema complications.

KL‐6 and SP‐D, as serum markers of ILD, are produced by type II alveolar epithelial cells.^33^, ^34^, ^35^ KL‐6 is a plasma membrane protein belonging to the MUC1 mucin family and SP‐D is a secreted glycoprotein with innate immunomodulatory properties.^36^ Although the levels of both are elevated in drug‐induced lung injury,^17^, ^18^, ^37^ only KL‐6 has been reported as a predictor of ICI‐induced pneumonitis.^38^ However, the patient population in this study, primarily receiving combined immunotherapy for lung cancer, differs from that of previous research, potentially explaining the variance in the predictive capacity of KL‐6. Elevated KL‐6 levels in lung adenocarcinoma, increasing with disease progression,^39^ may have contributed to its limited predictive value for severe pneumonitis in this study. Conversely, SP‐D expression decreases in lung cancer tissue (in both adenocarcinomas and SQ) compared with that in healthy tissue.^40^ In this study, the SP‐D levels were similar in adenocarcinoma and SQ (data not shown); we believe that the elevated SP‐D levels may reflect ILD in the surrounding or affected lung tissue. Consequently, SP‐D may serve as a potential prognostic factor in this context. We previously demonstrated an association between the development of severe irAEs and overall tumor burden in patients undergoing first‐line nivolumab plus ipilimumab treatment with or without chemotherapy.^22^ In the context of severe pneumonitis, DLCO and SP‐D, but not overall tumor burden, were identified as predictive factors, emphasizing the significance of comorbid ILD. Notably, in an observational study of nivolumab plus ipilimumab with treatment or without chemotherapy in Japan, the frequency of grade 3 or higher lung inflammation was 6.2%, with 98% of patients not having ILD at baseline.^41^ In contrast, in this study, we observed a 33.3% incidence of severe pneumonitis in patients with ILD before treatment initiation (7/21 patients), whereas patients without ILD (F score = 0) showed a lower incidence of 10.9% (6/55 patients). The occurrence of severe pneumonitis in patients with high tumor burden suggests that risk factors for pneumonitis may differ based on the presence or absence of ILD, warranting further validation.

In this study, severe pneumonitis was more frequent in SQ by histological type. Among the 37 patients with SQ, 11 (29.7%) exceeded the SP‐D cutoff value (≥103) and 10 (27.0%) exceeded the %DLCO cutoff value (≤71.1). Furthermore, five patients (13.5%) exceeded the cutoff levels of both. On the contrary, among 39 patients with nonsquamous cell carcinoma (NSQ), 10 (25.6%) exceeded the SP‐D cutoff level, 10 (25.6%) exceeded the %DLCO cutoff value, and five (12.8%) exceeded the cutoff levels of both. Although the number of patients exceeding the cutoffs for SP‐D and %DLCO was similar for patients with SQ and those with NSQ, 7/39 (17.9%) patients with NSQ had F score ≥1; whereas 14/37 (37.8%) patients with SQ had F score ≥1 with a higher rate of lung fibrosis. Although the degree of smoking was not precisely assessed in this study, it could be speculated that patients with SQ had poorer baseline lung conditions.

The JCOG 2007 phase III trial in Japan, comparing platinum‐based combination chemotherapy with nivolumab plus ipilimumab (NIVO + IPI arm) to platinum‐based combination chemotherapy with pembrolizumab in untreated advanced NSCLC,^42^ was terminated prematurely owing to 11 (7.4%) treatment‐related deaths in the NIVO + IPI arm, with four of these deaths attributed to pneumonitis. In our study, the incidence of grade 3 or higher pneumonitis in the NIVO + IPI and NIVO + IPI + chemo groups was similar at 17.1% (6/35 patients) and 17.1% (7/41 patients), respectively, with no heightened risk observed with the addition of chemotherapy. Although the high SP‐D level was a risk factor in both NIVO + IPI and NIVO + IPI + chemo treatments as determined using the risk factors used in the multivariate analysis for each regimen, the F score and %DLCO were associated with only NIVO + IPI + chemo treatment, suggesting that existing lung fibrosis might have more influence on severe pneumonitis development when chemotherapy is used in combination. Furthermore, eight of the 13 patients with severe pneumonitis (NIVO + IPI, 4 patients; NIVO + IPI + chemo, 4 patients) had other irAEs; among them, four patients (NIVO + IPI, 2 patients; NIVO + IPI + chemo, 2 patients) had grade 3 or higher irAEs (2 patients had skin disorders, 2 patients had liver dysfunction, 1 patient had renal dysfunction, 1 patient had cytokine release syndrome, and 1 patient had an infusion‐related reaction; duplicate irAEs for the same patient available). Severe pneumonitis with NIVO + IPI ± Ccemo might be complicated by multiple grade 3 or higher irAEs, and close attention should be paid to the patient's general condition at the onset of the disease. Therefore, continuous examination of risk factors for fatal irAEs is imperative.

Despite contributing valuable insights, this study had limitations. First, it was a single‐center retrospective observational study, potentially introducing bias in treatment and patient selection. Second, the short observation period might have led to an underestimation of late‐stage treatment irAEs. Finally, the small number of patients could introduce bias in the frequency and severity of irAEs.

In conclusion, our findings suggest that baseline SP‐D and %DLCO serve as potential risk factors for the development of severe pneumonitis in patients treated with first‐line nivolumab plus ipilimumab with/without chemotherapy. Vigilant monitoring of patients with high a SP‐D level or low %DLCO is recommended to prevent severe pneumonitis and facilitate early therapeutic intervention. Further studies are required to explore the predictive factors of nivolumab plus ipilimumab‐related pneumonitis in patients with both NSCLC and ILD.

## AUTHOR CONTRIBUTIONS

All authors reviewed the manuscript draft and revised it critically for intellectual content. All authors read and approved the final manuscript. All authors had full access to the data in the study and take responsibility for the integrity of the data and the accuracy of the data analysis. Conceptualization: Toshiyuki Sumi. Statistical analysis: Motoki Sekikawa, Yuta Koshino, Daiki Nagayama, Yuta Nagahisa, Keigo Matsuura, Naoki Shijubou, Koki Kamada, Keito Suzuki, Hiroki Watanabe, Koichi Osuda and Yuichi Yamada. Results interpretation: Yusuke Tanaka. Writing original draft: Toshiyuki Sumi. Supervision: Hirofumi Chiba.

## FUNDING INFORMATION

This research did not receive any specific grant from funding agencies in the public, commercial, or not‐for‐profit sectors.

## CONFLICT OF INTEREST STATEMENT

The authors declare no conflicts of interest.

## Supporting information


**Figure S1.** Definition of the computed tomography (CT) scoring system for fibrosis and emphysema with specific examples.
**Figure S2.** Details of pneumonitis and time to onset.
**Figure S3.** Progression‐free survival and overall survival corresponding to the distinct severity levels of pneumonitis.
**Figure S4.** Association between severe pneumonitis and surfactant protein D (SP‐D) level, percent predicted forced vital capacity (%DLCO), and overall tumor burden in patients with a fibrosis score of 0 (*n* = 55).

**Table S1.** Summary of the number and severity of immune‐related adverse events in patients treated with nivolumab plus ipilimumab with or without chemotherapy.

**Table S2.** Summary of the number and severity of immune‐related adverse events in patients treated with nivolumab plus ipilimumab with or without chemotherapy.

**Table S3.** Patient characteristics with and without pneumonia of any grade.

**Table S4.** Characteristics of patients with severe pneumonitis and mild pneumonitis.

## Data Availability

The datasets generated and/or analyzed in the current study are available from the corresponding author on reasonable request.
