# Supplementary material for: Risk factors for severe immune‐related pneumonitis after nivolumab plus ipilimumab therapy for non‐small cell lung cancer
Source: Thorac Cancer. 2024 Jun 3;15(20):1572–81. doi: 10.1111/1759-7714.15385 (PMC11246787; doi:10.1111/1759-7714.15385)

Supplementary Figure 1. Definition of the computed tomography (CT) scoring system for fibrosis and emphysema with specific examples.

Abbreviation: LAA; low-attenuation area.


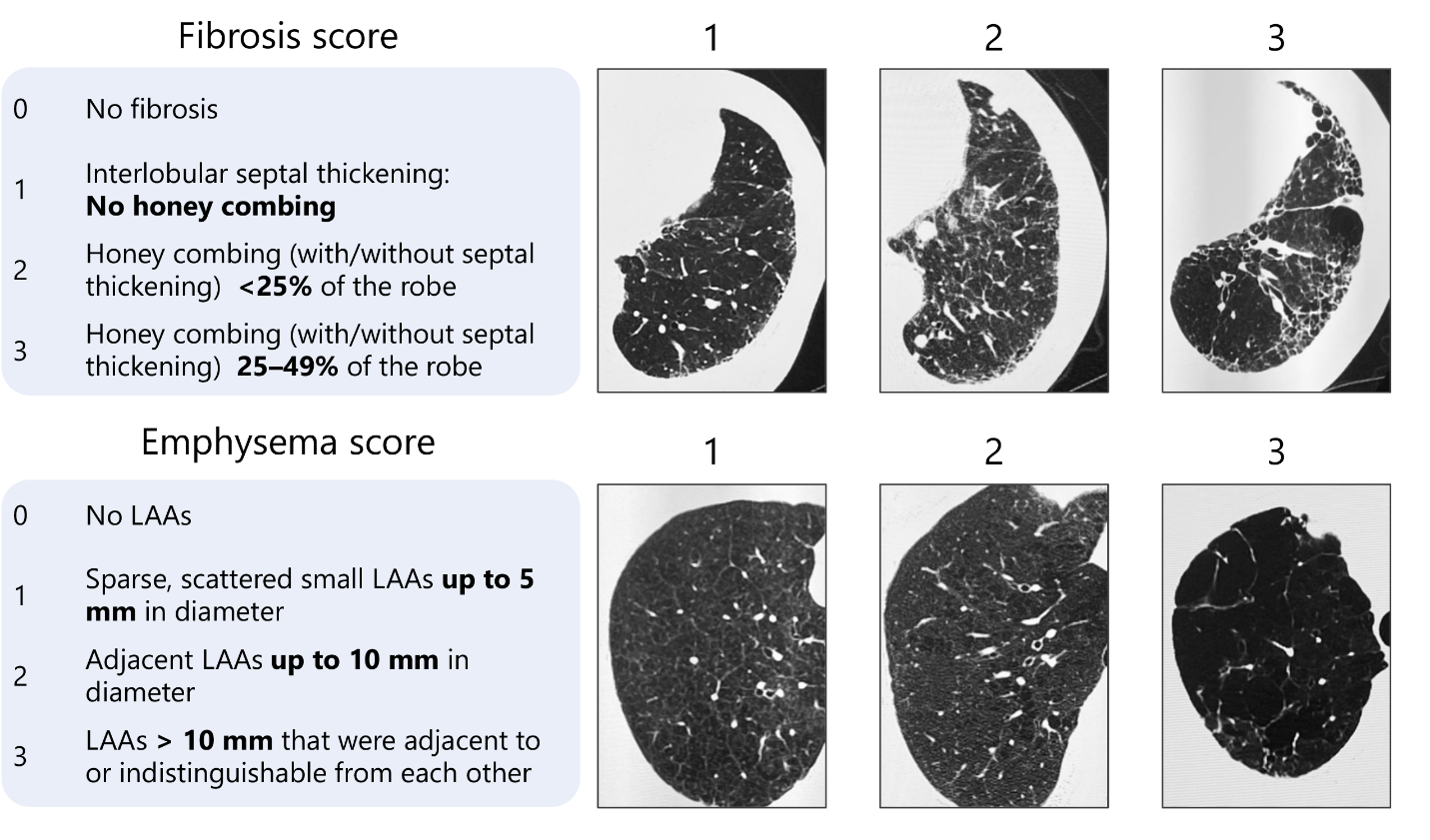


Supplementary Figure 2. Details of pneumonitis and time to onset.


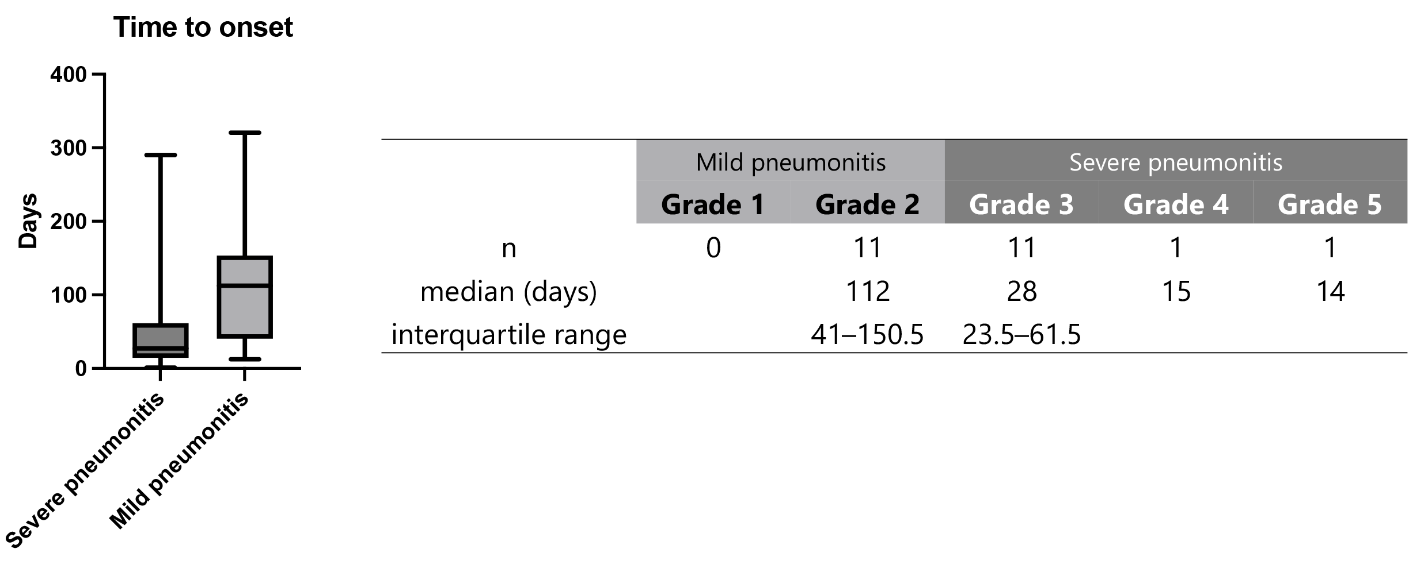


Supplementary Figure 3. Progression-free survival and overall survival corresponding to the distinct severity levels of pneumonitis.

Abbreviations: PFS, progression-free survival; OS, overall survival; CI, confidence interval.


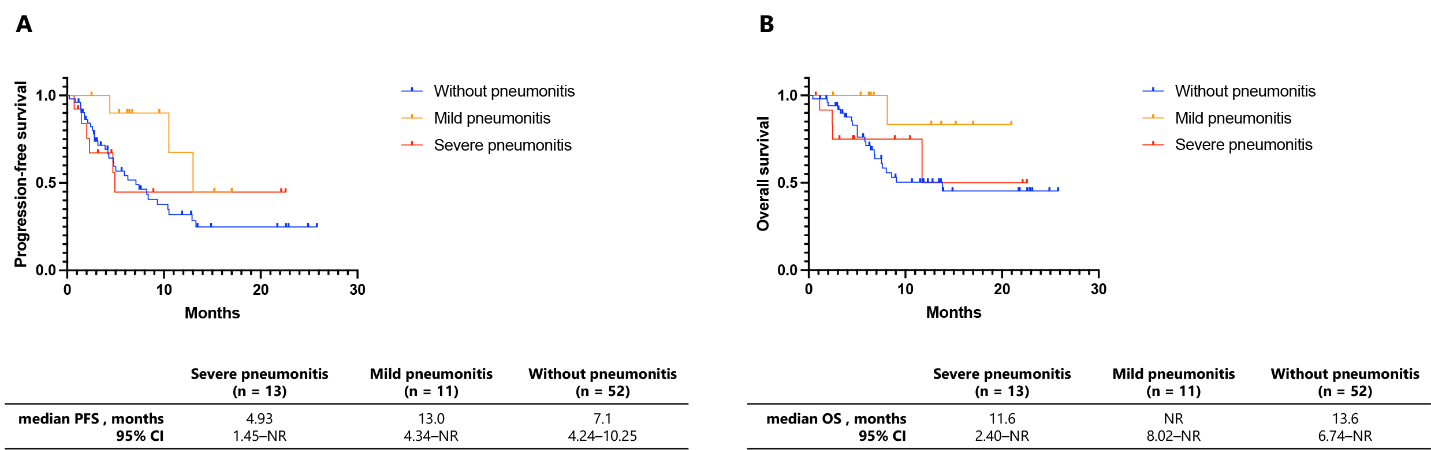


Supplementary Figure 4. Association between severe pneumonitis and surfactant protein D (SP-D) level, percent predicted forced vital capacity (%DLCO), and overall tumor burden in patients with a fibrosis score of 0 (n = 55).

Abbreviation: ns, not significant. *P < 0.05.


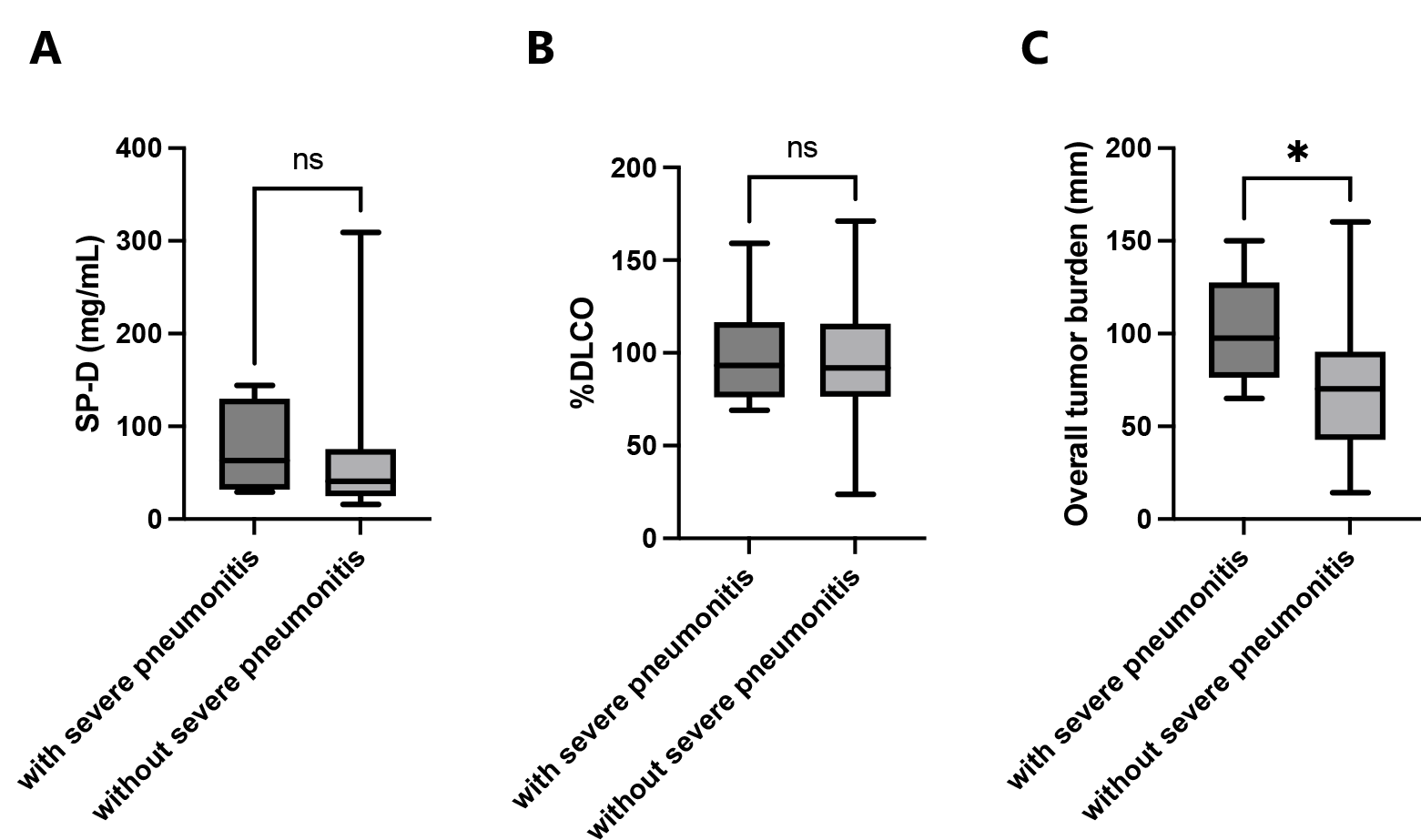

Supplement: Supplementary file 1 — Figure S1. Definition of the computed tomography (CT) scoring system for fibrosis and emphysema with specific examples. Figure S2. Details of pneumonitis and time to onset. Figure S3. Progression‐free survival and overall survival corresponding to the distinct severity levels of pneumonitis. Figure S4. Association between severe pneumonitis and surfactant protein D (SP‐D) level, percent predicted forced vital capacity (%DLCO), and overall tumor burden in patients with a fibrosis score of 0 (n = 55). [file TCA-15-1572-s001.docx]
