# Supplementary material for: Risk factors for severe immune‐related pneumonitis after nivolumab plus ipilimumab therapy for non‐small cell lung cancer
Source: Thorac Cancer. 2024 Jun 3;15(20):1572–81. doi: 10.1111/1759-7714.15385 (PMC11246787; doi:10.1111/1759-7714.15385)
Supplement: Supplementary file 2 — Table S1. Summary of the number and severity of immune‐related adverse events in patients treated with nivolumab plus ipilimumab with or without chemotherapy. [file TCA-15-1572-s004.docx]

## Supplementary Table 1. Summary of the number and severity of immune-related adverse events in patients treated with nivolumab plus ipilimumab with or without chemotherapy.

| **Treatment** | **Age** | **Smoking history** | **PS** | **Stage** | **PD-L1** | **Histology** | **Tumor burden** | **BOR** | **E score** | **F score** | **SP-D** | **KL-6** | **FEV1%** | **%VC** | **%DLCO** | **Pneumonitis** | **Time to onset**  **(days)** | **Rash** | **Endocrine disorder** | **Hepatitis** | **Colitis** |
| --- | --- | --- | --- | --- | --- | --- | --- | --- | --- | --- | --- | --- | --- | --- | --- | --- | --- | --- | --- | --- | --- |
| NIVO +IPI + chemo | 77 | former | 0 | III | 1–49% | SQ | 30 | PR | 1 | 1 | 158 | 861 | 65.42 | 94 | 77 | G2 | 12 | G2 | G2 |  |  |
| NIVO +IPI + chemo | 73 | former | 0 | Rec | ≥50% | AD | 80 | PR | 2 | 0 | 89.2 | 529 | 48.17 | 79.5 | 124 | G2 | 305 | G1 | G2 |  |  |
| NIVO +IPI + chemo | 74 | former | 0 | III | <1% | NOS | 45 | PR | 1 | 0 | 79.2 | 264 | 47.75 | 65.2 | 30.6 | G2 | 320 | G1 |  |  |  |
| NIVO +IPI + chemo | 76 | current | 0 | Rec | 1–49% | SQ | 20 | SD | 2 | 1 | 74.3 | 405 | 54.82 | 114.8 | 75.8 | G2 | 148 |  |  |  |  |
| NIVO +IPI | 75 | former | 0 | IV | <1% | AD | 22 | SD | 1 | 0 | 19.6 | 329 | 53.48 | 73.6 | 91 | G2 | 42 |  |  |  |  |
| NIVO +IPI + chemo | 64 | former | 1 | IV | ≥50% | AD | 20 | SD | 2 | 0 | 33.4 | 245 | 53.52 | 47.8 | 49.3 | G2 | 21 |  |  | G2 |  |
| NIVO +IPI | 80 | former | 1 | Rec | 1–49% | SQ | 60 | SD | 0 | 1 | 36.8 | 272 | 77.44 | 74.1 | 102.7 | G2 | 147 | G1 |  |  |  |
| NIVO +IPI + chemo | 78 | former | 0 | IV | <1% | AD | 95 | PR | 0 | 0 | 56.7 | 387 | 74.45 | 105 | 100 | G2 | 153 | G2 | G2 | G3 |  |
| NIVO +IPI | 81 | former | 1 | III | ≥50% | SQ | 100 | PR | 0 | 1 | 227 | 797 | 90.58 | 80.9 | 88.2 | G2 | 112 |  |  |  |  |
| NIVO +IPI | 72 | former | 1 | III | <1% | AD | 60 | PR | 1 | 0 | 49.2 | 386 | 77.48 | 71.2 | 61.7 | G2 | 84 | G2 |  |  |  |
| NIVO +IPI | 80 | former | 0 | IV | ≥50% | AD | 100 | PR | 2 | 1 | 130 | 934 | 74 | 55.4 | 58.8 | G2 | 40 |  |  |  |  |
| NIVO +IPI + chemo | 70 | former | 1 | III | 1–49% | SQ | 80 | PR | 0 | 0 | 60.6 | 337 | 65.25 | 86 | 102.4 | G3 | 10 | G3 | G2 | G3 | G2 |
| NIVO +IPI + chemo | 63 | current | 0 | III | ≥50% | SQ | 100 | PR | 0 | 0 | 144 | 388 | 52.15 | 94 | 159 | G3 | 290 | G2 | G2 |  |  |
| NIVO +IPI + chemo | 76 | former | 0 | III | 1–49% | SQ | 65 | PR | 1 | 0 | 29 | 562 | 52.94 | 88.9 | 94.5 | G3 | 32 |  |  |  |  |
| NIVO +IPI | 79 | former | 1 | IV | ≥50% | SQ | 150 | SD | 0 | 0 | 66 | 623 | 77.44 | 72.5 | 69.1 | G3 | 1 | G2 |  |  |  |
| NIVO +IPI | 79 | former | 1 | Rec | 1–49% | SQ | 120 | PD | 1 | 0 | 32.7 | 265 | 76.16 | 78.8 | 91.9 | G3 | 26 |  |  | G2 |  |
| NIVO +IPI | 82 | former | 0 | IV | 1–49% | SQ | 95 | PR | 2 | 0 | 125 | 341 | 87.4 | 88.3 | 78.5 | G3 | 121 |  |  | G3 |  |
| NIVO +IPI + chemo | 71 | former | 0 | IV | ≥50% | SQ | 70 | PR | 1 | 1 | 156 | 209 | 71.21 | 60.2 | 39.8 | G3 | 62 | G3 |  |  |  |
| NIVO +IPI | 70 | former | 0 | III | 1–49% | SQ | 30 | PR | 2 | 2 | 156 | 471 | 54.51 | 65.6 | 50.8 | G3 | 21 |  |  |  |  |
| NIVO +IPI + chemo | 61 | former | 1 | III | 1–49% | NOS | 70 | SD | 3 | 3 | 294 | 413 | 67.72 | 92 | 38.5 | G3 | 27 |  |  |  |  |
| NIVO +IPI | 79 | former | 1 | IV | 1–49% | SQ | 98 | PD | 2 | 3 | 108 | 468 | 75 | 89.5 | 40.1 | G3 | 61 |  |  |  |  |
| NIVO +IPI | 73 | former | 0 | IV | 1–49% | AD | 40 | PR | 2 | 1 | 108.2 | 536 | 56.11 | 107.4 | 58.8 | G3 | 28 |  |  |  |  |
| NIVO +IPI + chemo | 73 | current | 1 | IV | ≥50% | SQ | 170 | SD | 0 | 1 | 136 | 1580 | 80.61 | 61.3 | 52.4 | G4 | 15 |  |  |  |  |
| NIVO +IPI + chemo | 58 | former | 1 | IV | <1% | AD | 42 | PR | 2 | 1 | 158 | 468 | 83.28 | 87.7 | 54.4 | G5 | 14 |  |  |  |  |

NIVO, nivolumab; IPI, ipilimumab; chemo, chemotherapy; PS, performance status; BOR, best of response; Rec, recurrence; SQ, squamous cell carcinoma; AD, adenocarcinoma; NOS, not otherwise specified; PR, partial response; SD, stable disease; PD, progressive disease; SP-D, surfactant protein D; KL-6, Krebs von den Lungen-6; FEV1%, ％ percent predicted Forced Expiratory Volume in one second; %VC, vital capacity percentage; %DLCO, percent predicted diffusing capacity for carbon monoxide; G, grade.
