# Supplementary material for: Risk factors for severe immune‐related pneumonitis after nivolumab plus ipilimumab therapy for non‐small cell lung cancer
Source: Thorac Cancer. 2024 Jun 3;15(20):1572–81. doi: 10.1111/1759-7714.15385 (PMC11246787; doi:10.1111/1759-7714.15385)
Supplement: Supplementary file 3 — Table S2. Summary of the number and severity of immune‐related adverse events in patients treated with nivolumab plus ipilimumab with or without chemotherapy. [file TCA-15-1572-s002.docx]

## Supplementary Table 2. Summary of the number and severity of immune-related adverse events in patients treated with nivolumab plus ipilimumab with or without chemotherapy.

|  | **NIVO + IPI** | |  | **NIVO + IPI + Chemo** | |  |
| --- | --- | --- | --- | --- | --- | --- |
| **Factor** | **Patients with  severe pneumonitis  (n =6)** | **Patient without severe pneumonitis  (n = 29)** | **p value^a^** | **Patients with  severe pneumonitis  (n = 7)** | **Patient without severe pneumonitis (n =34)** | **p value^a^** |
| **Overall tumor burden ≥85 mm** | 4 | 7 | 0.063 | 2 | 14 | 0.69 |
| **F score ≥1** | 3 | 8 | 0.35 | 4 | 6 | 0.047 |
| **SQ** | 5 | 15 | 0.21 | 5 | 12 | 0.11 |
| **%DLCO ≤ 71.1** | 4 | 7 | 0.063 | 4 | 5 | 0.031 |
| **SP-D ≥ 103** | 5 | 7 | 0.012 | 5 | 5 | 0.006 |

^a^ Fisher’s exact tests
NIVO, nivolumab; IPI, ipilimumab; chemo, chemotherapy; F score, fibrosis score; SQ, squamous cell carcinoma; %DLCO, percent predicted diffusing capacity for carbon monoxide; SP-D, surfactant protein D
