# Supplementary material for: Risk factors for severe immune‐related pneumonitis after nivolumab plus ipilimumab therapy for non‐small cell lung cancer
Source: Thorac Cancer. 2024 Jun 3;15(20):1572–81. doi: 10.1111/1759-7714.15385 (PMC11246787; doi:10.1111/1759-7714.15385)
Supplement: Supplementary file 4 — Table S3. Patient characteristics with and without pneumonia of any grade. [file TCA-15-1572-s003.docx]

**Supplementary Table 3. Patient characteristics with and without pneumonia of any grade.**

| **Factor** |  | **Patient with any grade pneumonitis** | **Patient without pneumonitis** | **p value^a^** |
| --- | --- | --- | --- | --- |
| **n** |  | 24 | 52 |  |
| **Age (years)** | ≥75 | 12 | 21 | 0.46 |
|  | <75 | 12 | 31 |  |
| **Sex** | Female | 6 | 15 | 0.79 |
|  | Male | 18 | 37 |  |
| **PS** | 0 | 13 | 35 | 0.31 |
|  | ≥1 | 11 | 17 |  |
| **Stage** | III | 9 | 11 | 0.20 |
|  | IV | 11 | 35 |  |
|  | Rec | 4 | 6 |  |
| **PD-L1** | ≥50％ | 8 | 18 | 1 |
|  | 1–49% | 11 | 22 |  |
|  | <1% | 5 | 12 |  |
| **Regimen** | NIVO + IPI | 11 | 24 | 1 |
|  | NIVO + IPI + Chemo | 13 | 28 |  |
| **Histology** | NSQ | 10 | 29 | 0.33 |
|  | SQ | 14 | 23 |  |
| **E score≥1** |  | 17 | 21 | 0.025 |
| **F score≥1** |  | 12 | 9 | 0.005 |
| **%DLCO ≤ 71.1** |  | 12 | 8 | 0.004 |
| **SP-D ≥ 103** |  | 13 | 9 | 0.002 |
| **Overall tumor burden ≥85 mm** |  | 9 | 18 | 0.81 |

^a^ Fisher’s exact tests
ECOG PS, Eastern Cooperative Oncology Group performance status; PD-L1, programmed cell death ligand 1; NIVO, nivolumab; IPI, ipilimumab; chemo, chemotherapy; SQ, squamous cell carcinoma; E score, emphysema score; F score, fibrosis score; %DLCO, percent predicted diffusing capacity for carbon monoxide; SP-D, surfactant protein D
