# Supplementary material for: Risk factors for severe immune‐related pneumonitis after nivolumab plus ipilimumab therapy for non‐small cell lung cancer
Source: Thorac Cancer. 2024 Jun 3;15(20):1572–81. doi: 10.1111/1759-7714.15385 (PMC11246787; doi:10.1111/1759-7714.15385)
Supplement: Supplementary file 5 — Table S4. Characteristics of patients with severe pneumonitis and mild pneumonitis. [file TCA-15-1572-s005.docx]

## Supplementary Table 4. Characteristics of patients with severe pneumonitis and mild pneumonitis.

| **Factor** |  | **Patient with severe pneumonitis (≥ Grade 3)** | **Patient with mild pneumonitis (Grade 1 or 2)** | **p value^a^** |
| --- | --- | --- | --- | --- |
| **n** |  | 13 | 11 |  |
| **Age (years)** | ≥75 | 5 | 7 | 0.41 |
|  | <75 | 8 | 4 |  |
| **Sex** | Female | 3 | 3 | 1 |
|  | Male | 10 | 8 |  |
| **PS** | 0 | 6 | 7 | 0.44 |
|  | ≥1 | 7 | 4 |  |
| **Stage** | III | 5 | 4 | 0.48 |
|  | IV | 7 | 4 |  |
|  | Rec | 1 | 3 |  |
| **PD-L1** | ≥50％ | 4 | 4 | 0.15 |
|  | 1–49% | 8 | 3 |  |
|  | <1% | 1 | 4 |  |
| **Regimen** | NIVO + IPI | 6 | 5 | 1 |
|  | NIVO + IPI + Chemo | 7 | 6 |  |
| **Histology** | NSQ | 3 | 7 | 0.095 |
|  | SQ | 10 | 4 |  |
| **E score** |  | 9 | 8 | 1 |
| **F score** |  | 7 | 5 | 1 |
| **%DLCO ≤ 71.1** |  | 8 | 4 | 0.41 |
| **SP-D ≥ 103** |  | 10 | 3 | 0.038 |
| **Overall tumor burden ≥85 mm** |  | 6 | 3 | 0.42 |

^a^ Fisher’s exact tests
ECOG PS, Eastern Cooperative Oncology Group performance status; PD-L1, programmed cell death ligand 1; NIVO, nivolumab; IPI, ipilimumab; chemo, chemotherapy; SQ, squamous cell carcinoma; E score, emphysema score; F score, fibrosis score; %DLCO, percent predicted diffusing capacity for carbon monoxide; SP-D, surfactant protein D
